# Feature Papers in Vehicular Sensing

**DOI:** 10.3390/s23094495

**Published:** 2023-05-05

**Authors:** Felipe Jiménez

**Affiliations:** Instituto Universitario de Investigación del Automóvil (INSIA), Universidad Politécnica de Madrid, 28031 Madrid, Spain; felipe.jimenez@upm.es

This Special Issue compiles papers submitted by the Editorial Board Members of the Vehicular Sensing Section and outstanding scholars in this field. Our purpose is to present papers that typify the most insightful and influential original research and review the field’s key topics.

The scope of the Special Issue is quite broad, covering all ground transportation (such as road and rail), water transportation and air transportation. The presented topics range from physical models and components to advanced control systems for automated vehicles, including driver monitoring, comfort considerations, communication and cooperative systems, perception sensors and solutions to issues related to electric vehicles.

This Special Issue includes sixteen papers. The first and most extensive group focuses on road vehicles, and its papers can be classified into a number of subgroups.

First, Refs. [1,2,3] present advances related to vehicle dynamics and subsystems. In [1], an improved deep neural network modeling method is proposed based on two optimization algorithms, namely, the linear decreasing weight particle swarm optimization algorithm and the invasive weed optimization algorithm, to predict vehicles’ longitudinal–lateral responses. Ref. [2] deals with electrical power steering and anomaly detection. Since most current detection methods rely on prior knowledge, it is difficult to identify new or previously unknown anomalies. Thus, this paper proposes a deep learning approach, which consists of a two-stage process using an autoencoder and long short-term memory, to detect anomalies in electrical power steering sensor data. Finally, in order to adapt the development of vehicle automation technology to different levels of automation, the dynamic PWM coupling pressure regulation method is proposed in [3] based on an original automatic pressure-regulating valve for electronically controlled pneumatic brake systems.

A relevant issue regarding autonomous vehicles is the behavior of the driver when the road scenario causes a change in vehicle control. Ref. [4] presents a study of driver behavior during the transition between autonomous and manual modes using a CARLA simulator. In the study, the driver’s gaze focalization is registered and fused with the road’s semantic segmentation to track when and to what direction the user is paying attention.

The development and deployment of electric vehicles involve several considerations relating to conventional vehicles and traffic, and some uncertainties are proposed in [5,6]. For example, electromagnetic fields present problems that are specific to electric vehicles. Ref. [5] summarizes the measurement methods applicable to the complex electromagnetic fields of electric vehicles. The authors focus on the evaluation of drivers’ exposure to these electromagnetic fields and investigate the static magnetic field, the extremely low-frequency magnetic field and the radiofrequency electromagnetic field in relation to the use of these vehicles in urban transportation.

Furthermore, electric vehicle charging demand and charging station availability forecast challenging problems that could hinder the efficient deployment of these vehicles. Many existing deep learning methods have been proposed to address this issue; however, due to complex road network structures and complex external factors, commonly used algorithms can only extract information on the historical use of roads. To enhance prediction accuracy and interpretability, Ref. [6] proposes an Attribute-Augmented Spatiotemporal Graph Informer structure by combining the Graph Convolutional Network layer and the Informer layer to extract both the external and internal spatiotemporal dependence of relevant transportation data.

A particularly relevant subgroup of papers focused on vehicular communications in order to provide additional information about the transportation system. A comprehensive review of connected vehicle technologies is provided in [7], including fundamental challenges, state-of-the-art enabling technologies, innovative applications and potential opportunities that can benefit automakers, customers and businesses. Ref. [8] presents a solution based on vehicular communications for blind intersections with high accident rates due to the poor visibility of oncoming traffic. For this purpose, a Road-Side Unit-based Virtual Intersection Management over 802.11p system is used, and it is specifically prepared for rural areas.

Furthermore, the authors of [9] developed a new approach for modeling the physical layer of V2X communications that can be extended to a wide range of configurations without leading to extensive measurement or simulation campaigns at the link layer. Subsequently, the authors of [10] reveal the application of a Mobile Ad Hoc Network. This paper presents the development of a location-aided routing protocol in forest fire detection that is based on three criteria: the route length between nodes, temperature sensing and the number of packets within node buffers (i.e., route busyness).

Two rail transportation issues are discussed in [11,12]. Ref. [11] explores the influence of changes in suspension parameters on passengers’ comfort. The ride comfort of the vehicle was assessed based on the standard method, which involves calculating the mean comfort, while the stiffness and damping parameters of the primary and secondary suspension systems were changed at three levels, and the vehicle was operated on a real track.

Meanwhile, Ref. [12] presents perception problems in sensors. LiDAR sensors are required in vehicular applications, and their applications in vehicles such as trams are quite strict as long braking distances are common. The authors test these types of sensors under different weather conditions and employ fake targets between the sensor and the tracked vehicle.

The second group of papers is related to air transportation and focuses on unmanned aerial vehicles. A vision-based guidance technique with a log polynomial closing velocity controller is presented in [13]. The goal was to achieve faster and more accurate landing compared to traditional vertical landing approaches by avoiding external disturbances and localization errors, which make autonomous landings on a moving target more challenging. Ref. [14] presents the application of an uncrewed aerial vehicle coupled with a thermal infrared sensor to identify new seeps in the long-dormant volcanic area of Ciomadul (Romania).

Finally, the final group of papers considers water applications. Ref. [15] provides an overview of possible solutions for improving the performance of measurement and control processes in maritime engineering applications. Considering that images captured underwater usually suffer from low contrast and color distortions due to light scattering and attenuation, the last paper proposes an underwater image enhancement method based on histogram-equalization approximation using physics-based dichromatic modeling [16].
